# Overview of antiviral medications used in ophthalmology

**Published:** 2020-03-30

**Authors:** Jeremy Hoffman

**Affiliations:** 1 Clinical Research Fellow: International Centre for Eye Health, London School of Hygiene & Tropical Medicine, UK.

**As eye health professionals, we are fortunate to have a number of antiviral medications available in our armoury to treat a range of ophthalmic viral infections. This article provides an overview of what antiviral agents are available for these conditions, detailing their regimen and evidence that supports their use.**

**Figure:**
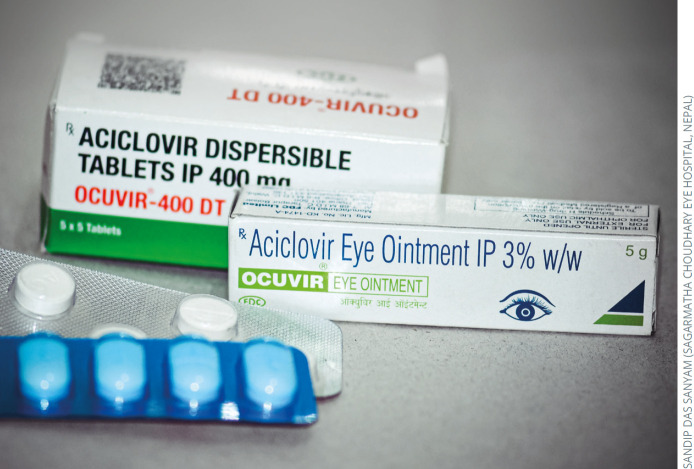
Aciclovir – either as topical eye ointment or systemic tablets – is still the first-line antiviral in the treatment of many viral eye diseases around the world, including here in Nepal.

Ophthalmic viral infections, particularly herpes simplex keratitis, have been at the forefront of the development of antiviral medications.

The discovery of the first targeted antiviral agent, in common with penicillin (the first antibiotic), owes much to serendipity. In 1959, William Prusoff developed idoxuridine (IDU) as a potential systemic anti-cancer agent. Idoxuridine proved to be too toxic as a systemic agent; however, its mechanism of action – selectively blocking DNA synthesis – proved to be a successful strategy in the topical treatment of herpes simplex virus (HSV), a DNA virus.^1^

**“By 1962, Herbert Kaufman had introduced idoxuridine (IDU) to the world as the first antiviral drug to successfully treat a human viral infection: herpes simplex keratitis.”**

By 1962, Herbert Kaufman had introduced idoxuridine (IDU) to the world as the first antiviral drug to successfully treat a human viral infection: herpes simplex keratitis.^2^ For the next decade, idoxuridine was the treatment of choice for epithelial herpes simplex keratitis. However, this was not perfect as it was frequently associated with toxicity, including superficial punctate keratopathy, chemical conjunctivitis, punctal occlusion and occasional serious hypersensitivity reactions. Idoxuridine was also unable to penetrate the corneal epithelium to treat stromal or endothelial keratitis.

With the advent of aciclovir in 1982, most herpetic ophthalmic infections became treatable, including those caused by herpes zoster.^3^ Since then, a number of newer antivirals with similar mechanisms of action have been developed, including ganciclovir, famciclovir, valaciclovir and valganciclovir, which work in a variety of ways to inhibit the synthesis of viral DNA and thereby stop viral replication. The addition of foscarnet, which acts as DNA polymerase inhibitor, has enabled ophthalmologists to add cytomegalovirus (CMV) retinitis to the list of treatable ophthalmic viral infections. However, despite a number of attempts and potential agents, there has been limited progress in developing antiviral treatment for adenovirus.

This article provides a summary of the current ophthalmic antiviral agents that are available to treat anterior and posterior segment viral infections.

## Antivirals acting on anterior segment viral infections

### Herpes simplex epithelial keratitis

Herpes simplex epithelial keratitis can be treated effectively with either topical aciclovir, ganciclovir or trifluridine, as detailed below (Table 1).

The choice of agent will depend on local availability, cost and patient factors.

Oral treatment may be preferable in patients who have ocular surface disease or a poor tear film.

Avoid systemic treatment in patients known to have poor renal function.

Vidarabine 3% ointment has been withdrawn from UK and US markets, but it may still be an option in some low- and middle-income countries. It can be used 5 times a day until the epithelium has healed.

**Table 1:** Antiviral treatment for herpes simplex epithelial keratitis

Drug	Dose / regimen	Comment
**Topical aciclovir (ACV)**	Aciclovir 3% ointment, five times a day for seven days, then three times a day for seven days^4^	Specific for viral-infected cells only; not available in USA and some supply issues elsewhere Resistance may be increasing in immunocompromised patients (10%)^5^
**Systemic aciclovir (ACV)**	Aciclovir 400 mg orally, five times a day for 7–10 days^4^	As effective as topical aciclovir^6^; toxicity is rare but requires normal renal function
**Topical ganciclovir (GCV)**	Ganciclovir 0.15%, 5 times daily until the ulcer has healed, then three times a day for seven days^4^,^7^	Increasing usage in UK and Europe due to aciclovir supply issues; as good as topical aciclovir for herpes simplex epithelial keratitis
**Topical trifluridine (TFT)**	Trifluridine 1% solution, 4–8 times a day^4^,^8^	First-line therapy in the USA; as effective as topical aciclovir
**Topical idoxuridine (IDU)**	Idoxuridine 0.5% ointment or IDU 1% solution, five times a day^1^,^4^	First topical antiviral; usage superseded by aciclovir, ganciclovir and trifluridine

### Herpes simplex stromal keratitis

Topical steroids in combination with either topical trifluridine **OR** topical aciclovir **OR** systemic aciclovir is better than treatment with antivirals alone (Table 2).

There may be some benefit of adding oral aciclovir to a course of topical steroids and topical anti-viral (trifluridine or aciclovir) for HSV-1 iritis.

At present there is little evidence for the use of ganciclovir 0.15% in the treatment of herpes simplex keratitis in combination with topical corticosteroids, and there are no randomised controlled trials comparing its efficacy to aciclovir or trifluridine. Despite this, it is likely to be of clinical value if alternatives are not available or tolerated.

**Table 2:** Antiviral treatment options for herpes simplex stromal keratitis. Note that these are all in addition to topical corticosteroids

Drug	Dose / regimen	Comment
**Topical aciclovir**	Aciclovir 3% ointment, five times a day whilst using topical corticosteroids	Useful as topical antiviral cover when using topical steroid. As effective as systemic aciclovir in conjunction with topical steroids for herpes simplex keratitis^6^
**Systemic aciclovir**	Aciclovir 400 mg orally, five times a day for ten weeks^9^,^10^	No additional benefit when added to topical trifluridine and topical corticosteroids for herpes simplex keratitis.^9^,^10^ There may be some benefit of adding oral aciclovir to topical trifluridine and topical corticosteroids in cases of HSV-1 iritis, although the study did not complete enrolment^11^ As effective as topical aciclovir in conjunction with topical steroids for herpes simplex keratitis^6^
**Topical trifluridine**	Trifluridine 1% 4–8 times a day for 3 weeks^9^,^10^	Useful as topical antiviral cover when treating herpes simplex keratitis with topical corticosteroids

### Prophylaxis / prevention strategies against recurrent HSV ocular infections

There is strong evidence from the Herpes Eye Disease II (HEDS II) study^12^ that prophylactic aciclovir 400 mg twice daily reduces the recurrence of potentially blinding herpes simplex stromal keratitis with a relative risk reduction of 50% (Table 3). It is important to measure renal function on an annual basis for patients who are on prophylactic aciclovir.

**Table 3:** Antiviral prophylaxis for the prevention of re-activation of HSV ocular infections

Drug	Dose / regimen	Comment
**Systemic aciclovir**	Aciclovir 400 mg orally twice a day	There is strong evidence^12^ that the use of prophylactic systemic aciclovir reduces the recurrence of: Any form of ocular HSV infection (19% aciclovir vs. 32% control; p < 0.001) Herpes simplex keratitis specifically (14% aciclovir vs. 28% control; p < 0.005)

### Herpes zoster ophthalmicus (varicella zoster virus)

Oral aciclovir 800 mg five times a day for seven days remains the most widely used, affordable and available treatment for herpes zoster. It is important to start this as soon as possible; there is limited evidence of its efficacy if started more than 72 hours after the rash develops for non-ocular involvement (Table 4).

Topical antivirals (aciclovir or ganciclovir) may have a supplementary role in the presence of dendritic or pseudo-dendritic keratitis but should not be used on their own.

Topical corticosteroids should be used in the presence of stromal keratitis or iritis, and can be started in the presence of pseudo-dendrites.

Newer oral antivirals such as valaciclovir or famciclovir have better bioavailability and dosing regimes, but availability and cost remain barriers to their use globally.

**Table 4:** Antiviral treatment options for herpes zoster ophthalmicus (caused by varicella zoster virus)

Drug	Dose / regimen	Comments and evidence for use
**Systemic aciclovir**	Aciclovir 800 mg orally five times a day for seven days	For non-ocular involvement at onset, treatment must start within 72 hours of onset of blisters in order to alter disease course^13^ For ocular disease, start as soon as possible Antiviral treatment reduces the risk of chronic ocular complications from 30% to 20%^14^ Reduces duration of pain due to post-herpetic neuralgia (PHN) by 50% although no reduction in risk of developing PHN^14^ Consider using intravenous aciclovir in HIV infected patients due to the risk of disseminated varicella zoster virus infection
**Topical aciclovir**	Aciclovir 3% ointment, five times a day for seven days, then twice a day until dendrites have resolved	Use in the presence of dendritic keratitis but only in addition to systemic antiviral treatment. Add a topical steroid if there is stromal disease or keratitis^15^
**Topical ganciclovir**	Ganciclovir 0.15%, five times a day until the ulcer has healed	Use in the presence of dendritic keratitis only in addition to systemic antiviral treatment. Add a topical steroid if there is stromal disease or keratitis
**Systemic valaciclovir**	1 g orally three times a day for seven days	Alternative to aciclovir. Higher serum concentrations following oral administration, due to better bioavailability, means more convenient dosing (3 times a day vs 5 times a day) Potentially better than aciclovir in reducing acute pain^16^
**Systemic famciclovir**	500 mg orally three times a day for seven days	Alternative treatment options to aciclovir. Higher serum concentrations following oral administration, due to better bioavailability, means more convenient dosing (3 times a day vs 5 times a day) Potentially better than aciclovir in reducing acute pain^17^

### Adenovirus ocular infections

There is currently no licensed antiviral agent for the treatment of adenoviral infections. However, a recent phase 2 randomised-controlled trial suggests benefit in using a combination of povidone-iodine 0.6% and dexamethasone 0.1% (four times a day to both eyes for five days).^18^ The follow-up period for this study was short (only 12 days). The combination treatment is not currently available, nor licenced. The effect of the topical steroid treatment on intraocular pressure also has to be considered.^19^

Povidone-iodine alone may be effective; however, further studies are required to assess this.

Other agents under investigation include ganciclovir 0.15% gel.

## Antivirals acting on posterior segment viral infections

### CMV retinitis

#### Systemic treatments

All systemic treatment must be administered in partnership with an HIV or infectious disease physician.

Oral valganciclovir is effective and easily administered.^20^ However, it is expensive and not always available. Care must be taken to monitor renal function and full blood counts.

Alternative systemic treatments include systemic intravenous ganciclovir (Table 5).

Foscarnet^21^ is very rarely used now because of the advent of highly active antiretroviral therapy (HAART) and the need for daily two-hour infusions via indwelling catheter.

#### Intravitreal treatment

Intravitreal ganciclovir injection is a very useful option as it can be done as an outpatient and gives high levels of concentration of the drug where it is needed. However, it does not protect the other eye or protect against systemic CMV. It should be given as adjuvant therapy if there is infection within 1 disc diameter of the fovea or optic disc.

**Table 5:** Antiviral treatment options for CMV retinitis

Drug	Dose / regimen	Comments and evidence for use
**Systemic oral valganciclovir**	Induction dose: 900 mg orally twice a day for 14–21 days, followed by maintenance dose of 900 mg orally once a day until CD4 count normalises	Need to monitor full blood count and renal function due to potential bone marrow suppression and renal toxicity; expensive As effective as intravenous ganciclovir for induction and long-term therapy for CMV retinitis in HIV patients^20^
**Systemic intravenous ganciclovir**	Induction dose: 5 mg/kg/dose every twelve hours for 1–21 days, followed by a maintenance dose of 5 mg/kg once a day until CD4 count normalises	Need to monitor full blood count and renal function due to potential bone marrow suppression and renal toxicity. Requires hospital attendance/admission for intravenous administration. First generation antiviral; effective^22^
**Intravitreal ganciclovir**	2.5 mg in 0.1 ml once a week	An alternative if systemic valganciclovir or ganciclovir is not available or too expensive^23^ All patients who have infection within 1-disc diameter of the fovea or optic disc should receive intravitreal injections Inexpensive, can be given as an outpatient Risk of endophthalmitis following intravitreal injection

### Acute retinal necrosis, progressive outer retinal necrosis

Although acute retinal necrosis (ARN) and progressive outer retinal necrosis (PORN) are different clinical entities (ARN affecting immunocompetent individuals and PORN affecting immunocompromised individuals). Both are caused by varicella zoster virus and, to a lesser extent, by HSV-1 and HSV-2.

The antivirals used to treat ARN and PORN are similar. The treatment options are:

- Initially, intravenous aciclovir (10 mg/kg three times a day) for 5–10 days, then oral aciclovir (800 mg five times a day) for 4–6 weeks
- Valaciclovir 2 g four times a day for 7–10 days, then oral valaciclovir 1 g three times a day for six weeks; this option can be given as an outpatient.

Either of these options can be combined with intravitreal foscarnet 2.4 mg (if available) which has been shown to reduce the risk of retinal detachment and hastens viral inactivity.^24^
